# Extent of reoperation predicts survival in recurrent IDH-wildtype glioblastoma based on institutional data and individual patient data meta analysis

**DOI:** 10.1007/s12672-025-03928-8

**Published:** 2025-11-13

**Authors:** Alim Emre Basaran, Ann-Catalin Wellkisch, Erdem Güresir, Johannes Wach

**Affiliations:** 1 Department of Neurosurgery, University Hospital Leipzig, Leipzig University, Liebigstr. 20, 04103 Leipzig, Germany; 2 Comprehensive Cancer Center Central Germany, Partner Site Leipzig, 04103 Leipzig, Germany

**Keywords:** Recurrent IDH-wildtype glioblastoma, Extent of resection, Survival outcomes, Surgical re-resection

## Abstract

**Background:**

Glioblastoma (GB) is the most aggressive brain tumor, characterized by rapid progression and poor prognosis. Despite initial multimodal treatment options, therapeutic options become more limited upon recurrence. Consequently, recurrent IDH-wildtype GB is associated with poor survival outcomes, with limited data to guide optimal therapeutic strategies. This study presents the largest meta-analysis to date, pooling institutional data with individual patient data (IPD), addressing progression-free survival (PFS) and overall survival (OS) from timepoint after re-resection.

**Methods:**

Institutional data and data from literature (2016–2024) were analyzed to evaluate PFS and OS in relation to the extent of resection (EoR). Survival data from identified studies were extracted from Kaplan-Meier curves with the R package IPDfromKM. Additionally, a retrospective analysis of institutional data was conducted, assessing for PFS and OS in 53 patients. EoR was dichotomized as suggested by the RANO group into residual contrast-enhancing tumor volume (CE-RTV) ≤ 1 cm^3^ or > 1cm^3^.

**Results:**

A total of 442 IPD were included in this meta-analysis. Among them, 331 patients (74.9%) underwent neurosurgical treatment with CE-RTV ≤ 1cm^3^, while 111 patients (25.1%) had CE-RTV > 1 cm^3^. Pooled analysis indicated a significant reduction in OS after re-resection with CE-RTV > 1 cm^3^ compared to CE-RTV ≤ 1cm^3^ (HR: 1.731, 95% confidence interval (CI): 1.342–2.234, *p* < 0.0001). While re-resection with CE-RTV ≤ 1cm^3^ was associated with longer OS (14.4 months) compared to CE-RTV > 1 cm^3^ (8.8 months) (*p* < 0.0001), PFS did not differ between the two groups (CE-RTV ≤ 1 cm^3^: 7.2 months compared to CE-RTV > 1cm^3^: 5.8 months) (*p* = 0.76).

**Conclusion:**

Across pooled IPD, maximal safe resection at re-resection operationalized as GTR or RANO class 1 and 2 was significantly associated with longer overall survival (OS). Where volumetric assessment is available, achieving a postoperative CE-RTV ≤ 1 cm^3^ may be a reasonable pragmatic target, however this threshold was not directly measured in all included cohorts and should be interpreted as hypothesis-generating.

**Graphical abstract:**

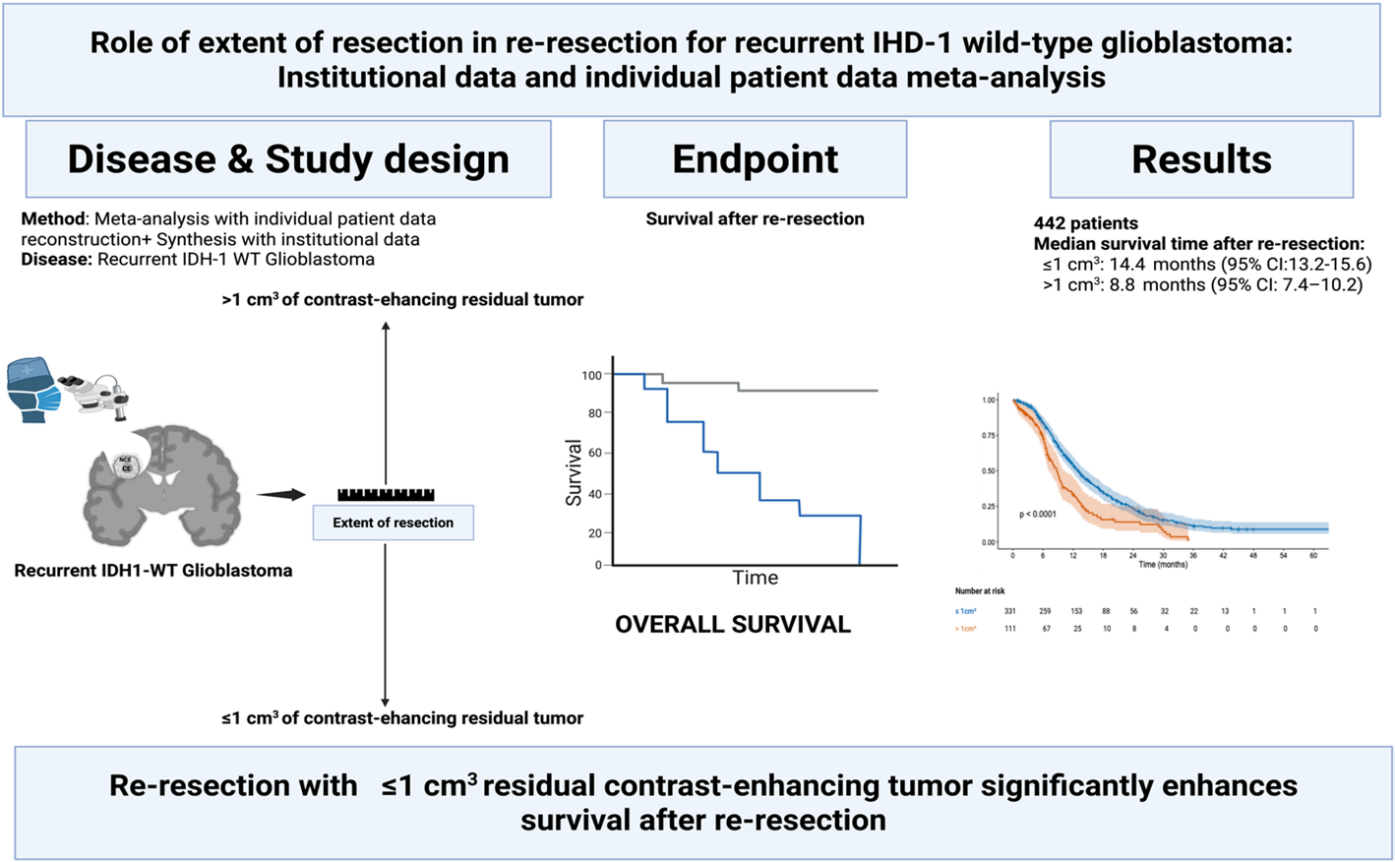

**Supplementary Information:**

The online version contains supplementary material available at 10.1007/s12672-025-03928-8.

## Introduction

Glioblastoma (GB) is the most common and malignant primary brain tumor characterized by rapid progression and a poor prognosis [1, 2]. Despite multimodal treatment strategies including neurosurgical tumor resection, radiotherapy and chemotherapy tumor relapse is unavoidable in most cases [3]. Hence, recurrent isocitrate-dehydrogenase (IDH) wildtype GB remains a major therapeutic challenge in this patient cohort. To date, no standardized treatment guidelines for recurrent GB exist and survival outcomes remain poor [4, 5]. 

Neurosurgical tumor resection is a disease modifying key component in primary GB treatment, where gross total resection (GTR) is associated with statistically significant prolongation of overall survival (OS) compared to subtotal resection (STR) [6, 7]. Additionally, the extent of resection (EoR) is influenced by several factors e.g., tumor localization, neurological status, and preoperative Karnofsky Performance Scale (KPS) [8, 9]. Despite the role of re-resection in recurrent GB has been investigated in previous prospective trials, the ongoing molecular revisions of GB classification necessitate revalidation of the role of surgery [10]. 

According to the Response Assessment in Neuro-Oncology (RANO) classification for EoR, residual contrast-enhanced residual tumor volume (CE-RTV) of ≤ 1 cm³ is prognostic regarding survival after re-resection. However, these findings lack broader validation. By integrating secondary pooled individual patient data with original institutional data, the present study aims to strengthen the evidence base, ensuring a more comprehensive assessment of re-resection outcomes and refining prognostic stratification in recurrent GB.

Against this backdrop, the primary aim of present investigation is to evaluate the impact of EoR in re-resection for IDH-wildtype GB and the association between EoR and Survival after re-resection.

## Materials and methods

### Search strategy and data collection

The present meta-analysis was conducted according to the Preferred Reporting Items for Systematic Reviews (PRISMA) guidelines and was prospectively registered in the International Prospective Register of Systematic Reviews (PROSPERO, ID: CRD420250654313) [11]. IPD were extracted from Pubmed, Google Scholar and Cochrane from January 1, 2016, to December 31, 2024. Following search items were used: (Glioblastoma OR GBM) AND (recurrent OR recurrence) AND (surgery OR reoperation OR resection) NOT (“Case Reports”[Publication Type]) NOT (in vitro OR in vivo OR “animal study”).

For institutional original data, we conducted a retrospective screening of patients who underwent neurosurgical re-resection for recurrent IDH-wildtype GB, between January 1, 2016, and December 31, 2024.

### Data extraction and individual patient data reconstruction

Two authors (AEB, JW) independently extracted the following data from the publications: country, year of publication, number of patients, sex, age, neuroanatomical location, MGMT status, IDH-1/2 status, EoR from first and second surgery, adjuvant therapy, OS and PFS after re resection. To derive individual patient data (IPD) for survival analysis, Kaplan-Meier survival curves and number-at-risk tables from Straube et al. [12], Kim et al. [13], Bao et al. [14] and Karschnia et al. [15] were extracted using Digizelt software (Version 2.5.10, for macOS) and were reconstructed with R package IPDfromKM [16, 17]. We harmonized EoR categories across studies to enable pooling. Karschnia et al. [12, 15] and our institutional cohort provided RANO-resect classes or volumetric CE-RTV and served as anchors. Straube et al. [12] reported GTR, which we mapped to CE-RTV ≤ 1 cm³. Bao et al. [14] restricted inclusion to patients who underwent GTR at second surgery but did not report postoperative CE-RTV volumes; these cases were mapped to CE-RTV ≤ 1 cm³ for harmonization. Kim et al. assessed EoR on early postoperative (48–72 h) MRI and defined GTR as >99% removal; while compatible with near-total resection, this definition does not guarantee CE-RTV ≤ 1 cm³ in hypothetical ≥ 100 cm³ baseline tumors. Accordingly, in Kim et al. [13] we mapped GTR to ≤ 1 cm³ and non-GTR to >1 cm³, and we explicitly acknowledge potential non-differential misclassification. Where original sources used GTR/STR, pooled results are reported in CE-RTV categories (≤ 1 cm³ vs. >1 cm³) to align with RANO prognostic strata. For consistency, GTR was mapped to CE-RTV ≤ 1 cm³ and non-GTR to >1 cm³ at the time of each surgery, first resection and re-resection refer to early postoperative volumes. We included only patients for whom Kaplan-Meier curves with number-at-risk allowed IPD reconstruction and for whom EoR at re-resection was explicitly documented. Patients were excluded if IPD could not be reconstructed, if EoR at re-resection could not be mapped to these categories, if IDH-wildtype status or age ≥ 18 years was not satisfied, or if potential duplicates across reports were identified.

The present institutional original data included neuropathologically diagnosed IDH-wildtype GB based on the 2021 World Health Organization (WHO) classification [18]. The Inclusion criteria were age of 18 years or older at time of surgery, a neuropathologically confirmed wild-type GB and the distinction between < 1 cm^3^ or >1 cm^3^ CE-RTV for EoR stratification. The preoperative and postoperative tumor volumes were measured using the Smartbrush tool of semi-automatic segmentation software (BrainLAB^®^, Munich, Germany). Stratification was performed based on a CE residual tumor volume of ≤ 1 cm³ and >1 cm³. For the first resection and for the re-resection, CE-RTV was quantified on the early postoperative contrast enhanced T1-weighted MRI (typically within 24–72 h after surgery). Throughout the manuscript we report EoR using CE-RTV categories (≤ 1 cm³ vs. >1 cm³). Where the original source or a prior section uses GTR/STR, we consider GTR ≙ CE-RTV ≤ 1 cm³ and STR ≙ CE-RTV >1 cm³. Across the institutional cohort, GTR/STR were defined as CE-RTV ≤ 1 cm³ vs. >1 cm³ at each surgery. References to first resection CE-RTV denote immediate postoperative volumes after the initial surgery, references to re-resection CE-RTV denote immediate postoperative volumes after the second surgery. All re-resections in our institutional series were performed on recurrences with measurable contrast enhancement on preoperative T1-weighted post-contrast MRI. No purely non-contrast-enhancing recurrences underwent re-resection during the study period. For mixed lesions with minimal or patchy enhancement, EoR classification was based strictly on the absolute CE-RTV measured on the early postoperative MRI scan. Non-contrast-enhancing abnormalies was not used for EoR categorization.

In cases of discrepancies, consensus was achieved through re-assessment or by consulting the third author (EG). For a detailed overview of the study characteristics, Table 1 provides a detailed summary of the studies.

**Table 1:** Study-level summary of re-resected cohorts and their eligibility for the pooled individual patient data (IPD) analyses

Study	Country	*n* reported (re-resected)	*n* included for OS	*n* included for PFS	Sex (M: F)	Age, mean (± SD)	Neuroanatomical location	EoR after re-Resection	EoR first resection	MGMT promotor	Adjuvant therapy after re-resection
Straube et al. [16], 2024	Germany	44	33		n.a.	n.a.	n.a.	33 (100%) GTR	n.a.	n.a.	33 (100%) Radiochemotherapy
Kim et al. [11], 2024	South Korea	112	47		n.a.	n.a.	n.a.	32 (68.1%) GTR	n.a.	n.a.	47 (100%) Radiochemotherapy
Bao et al. [17], 2024	China	34	16	17	9:8	43.9 ± 11.6	Insula (1) Parietal (1) Subcortex (1) Occipital (1) Frontal (10) Frontotemporal (3)	17 (100%) GTR	17 (100%) GTR	10 (58.2%) Me 7 (41.8%) Un	17 (100%) Radiochemotherapy
Karschnia et al. [10], 2023	Germany	307	307		Ratio 1:0.6	57.1 ± 1	(sub-)cortical 51 (85.0%) deep-seated 4 (6.7%) multifocal 5 (8.3%) local 50 (83.3%) distant 10 (16.7%) dominant 32 (53.3%) (sub-)cortical 117 (74.5%) deep-seated 28 (17.8%) multifocal 12 (7.6%) local 138 (87.9%) distant 19 (12.1%) dominant 76 (48.4%) (sub)-cortical 66 (73.3%) deep-seated 11 (12.2%) multifocal 13 (14.4%) local 86 (95.6%) distant 4 (4.4%) dominant 46 (51.1%)	RANO 1 60 (19.54%) RANO 2 157 (51.14%) RANO 3 90 (29.32%)	22 (36.7%) RANO 1 27 (45.0%) RANO 2 9 (15.0%) RANO 3 0 RANO 4 2 (3.3%) n.a. 22 (14.0%) RANO 1 70 (44.6%) RANO 2 19 (12.1%) RANO 3 0 RANO 4 46 (29.3%) n.a. 13 (14.4%) RANO 1 36 (40.0%) RANO 2 22 (24.4%) RANO 3 0 RANO 4 19 (21.1%) n.a.	29 (48.3%) Me 28 (46.7%) Un 3 (5.0%) n.a. 48 (30.6%) Me 57 (36.3%) Un 52 (33.1%) n.a. 31 (34.4%) Me 36 (40.0%) Un 23 (25.6%) n.a.	9 (15.0%) Radiochemotherapy 7 (11.7%) Radiotherapy alone 30 (50%) Chemotherapy alone 10 (16.7%) Surgery alone 2 (3.3%) Bevacizumab 1 (1.7%) Experimental agents 1 (1.7%) n.a. 23 (14.7%) Radiochemotherapy 19 (12.1%) Radiotherapy alone 79 (50.3%) Chemotherapy alone 19 (12.1%) Surgery alone 6 (3.8%) Bevacizumab 7 (4.5%) Experimental agents 4 (2.6%) n.a. 14 (15.6%) Radiochemotherapy 12 (13.3%) Radiotherapy alone 36 (40.0%) Chemotherapy alone 18 (20.0%) Surgery alone 3 (3.3%) Bevacizumab 3 (3.3.%) Experimental agents 4 (4.4%) n.a.

n reported = all re-resected patients as reported by the source; n included OS-IPD/PFS-IPD = patients meeting all pre-specified criteria for inclusion in our pooled OS and/or PFS IPD datasets. Differences between n reported and n_ ncluded reflect methodological, pre-specified filters: (1) availability of Kaplan–Meier curves with number-at-risk enabling IPD reconstruction; (2) explicit extent-of-resection at re-resection mapped to, (3) histomolecular eligibility (IDH-wild-type) and age ≥ 18; and (4) de-duplication across reports. For Bao et al., the IORT arm (*n* = 17) was excluded by protocol; the surgery-only arm (n for OS = 17 and n for PFS = 16) contributed to OS and uniquely PFS IPD. Because of censoring patterns, lost-to-follow-up, or incomplete reporting, a subset of reported cases could not be reconstructed robustly from published KM curves and were not included

### Study design and ethics

We retrospectively identified all consecutive patients with recurrent IDH-wildtype glioblastoma who underwent neurosurgical re-resection at our institution between 1 January 2016 and 31 December 2024. Eligibility criteria included age ≥ 18 years, neuropathological confirmation of IDH-wildtype GB, and availability of an early postoperative T1-weighted contrast-enhanced MRI suitable for CE-RTV quantification. No a priori exclusions were applied for multifocal disease or Karnofsky Performance Status; eligibility for surgery was determined by the multidisciplinary tumor board. Cases lacking an evaluable early postoperative CE-MRI were excluded; no purely non-enhancing recurrences underwent re-resection in this interval. The study was approved by the Ethics Committee of the Medical University of Leipzig (approval 144/08-ek), which waived the requirement for written informed consent for this retrospective analysis of de-identified routine clinical data.

### Inclusion criteria

Only studies published in English were considered for selection. A minimum of three cases with follow-up data on PFS or OS, histopathologically confirmed wild-type GB and patients older than 18 years were required for inclusion.

### Quality assessment

To assess study quality and the risk of bias, the NIH Quality Assessment Tool (NIH-QAT) was applied, providing a standardized framework for identifying strengths and weaknesses in the research design [19]. 

### Statistical analysis

Kaplan-Meier curves for OS and PFS after re-resection were created using pooled IPD, with categorizing CE-RTV as ≤ 1 cm³ vs. >1 cm³. Significant differences in OS and PFS between groups were analyzed using the log-rank test. Additionally, a Cox proportional hazards regression model was applied to estimate the Hazard Ratio (HR) and its 95% confidence interval (CI). All statistical analyses were performed with R software (Version 4.3.1) using the Survival and Survminer packages, with significance defined at *p* < 0.05. Overall survival and PFS were evaluated using Kaplan–Meier curves by EoR and a Cox proportional-hazards model with EoR as the sole predictor (CE-RTV ≤ 1 cm³ vs. > 1 cm³). Survival analyses were restricted to extent of resection (EoR). Across the reconstructed external datasets, individual-level information on MGMT promoter methylation, tumor location, interval to re-resection, and post-re-resection therapy was not consistently available, and none of the contributing studies published Kaplan–Meier curves with number-at-risk stratified by EoR in combination with these variables. It was therefore not possible to perform survival modeling that incorporated these additional factors.

## Results

### Search results and included studies

**Fig. 1:**
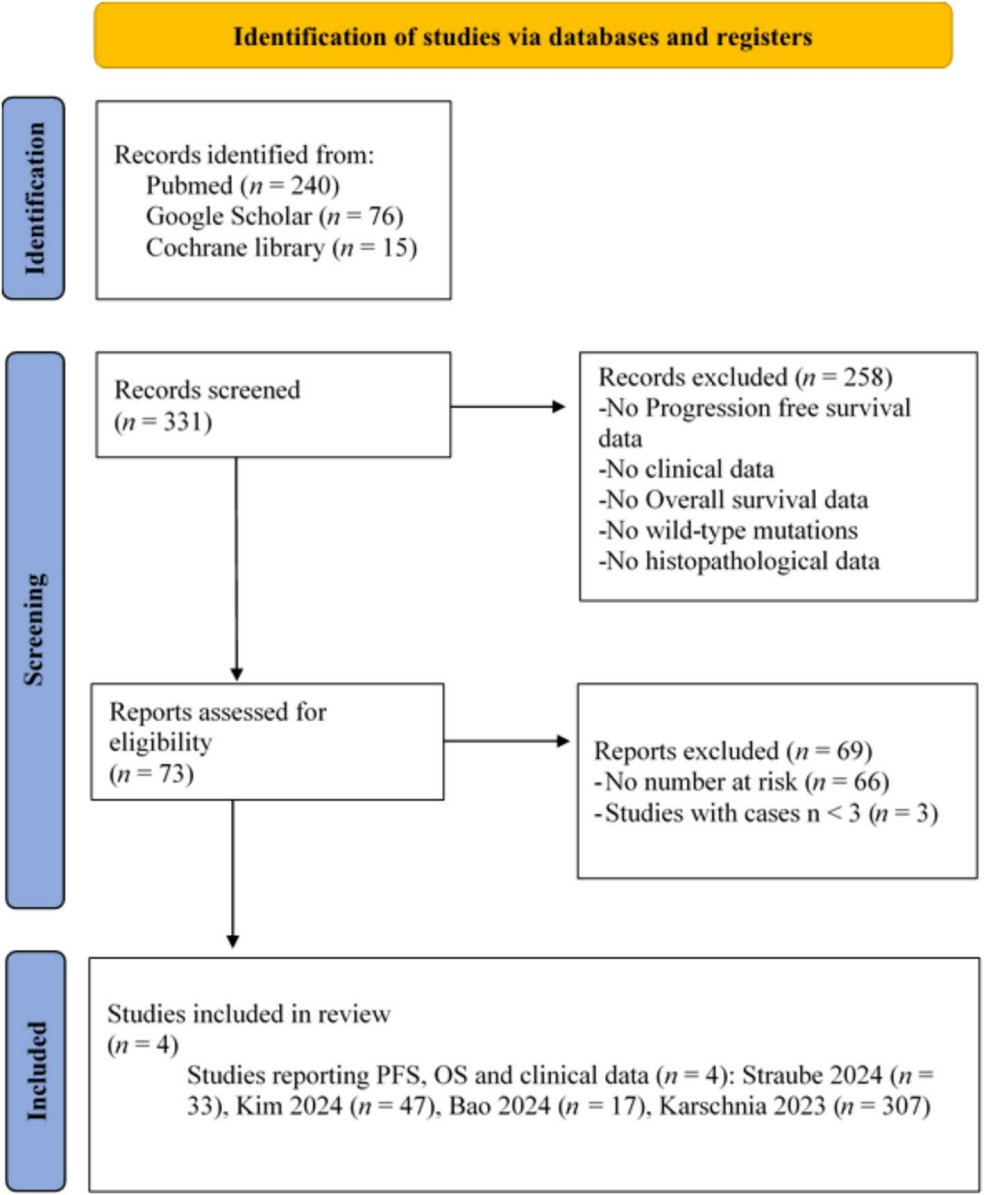
PRISMA flowchart study selection

In the initial search in Pubmed, Google Scholar and Cochrane library, we identified 331 studies. Of these, 258 studies were excluded due the absence of PFS data, clinical data, OS data, wild-type mutations, or histopathological data. The remaining 73 studies underwent a detailed review and analysis. Of these 73 studies, 66 studies were also excluded due the lack of number at risk data and three additional studies were excluded because they included fewer than three patients. After applying the inclusion criteria, four studies remained for the meta-analysis. The selected studies were published between 2023 and 2024 and included two studies from Germany, one from South Korea and one from China. All studies provided information about EoR and adjuvant therapy. Additionally, two studies reported neuroanatomical location and one study included information on EoR at the first surgery. After applying all eligibility criteria and harmonizing EoR at re-resection, the OS IPD dataset comprised 442 patients (including 53 institutional cases). For PFS, individual-level EoR-stratified data were available for 70 patients (institutional cohort plus Bao et al.). The PRISMA flow diagram and Supplementary Table S1 detail per-study exclusions (e.g., absence of number-at-risk tables, no EoR mapping at re-resection, ineligible histomolecular profile, or potential duplicates). For a detailed workflow and study characteristics see Table 1; Fig. 1.

### Institutional patient data

In the institutional cohort (*n* = 53), after re-resection, 32/53 (60.4%) had CE-RTV ≤ 1 cm³ and 21/53 (39.6%) had CE-RTV > 1 cm³ on the early postoperative MRI. At the first resection, 34/53 (64.2%) had CE-RTV ≤ 1 cm³ and 19/53 (35.8%) had CE-RTV > 1 cm³ on the early postoperative MRI. These proportions summarize each time point separately and do not represent within-patient transitions; differences between the two distributions reflect interval progression between surgeries and the extent achievable at recurrence. Furthermore, adjuvant therapy was administered as radiochemotherapy in 30 patients (56.6%), radiotherapy alone in 4 patients (7.5%) and chemotherapy alone in 2 patients (3.8%), while 17 patients (32.1%) did not receive any therapy after re-resection. Among the 32 patients who received chemotherapy 18 patients (56.3%) were treated with a combination regimen of temozolomide (TMZ) plus lomustine (CCNU) and 14 patients (43.8%) received temozolomide monotherapy. All 53 re-resected recurrences demonstrated measurable contrast enhancement preoperatively. Further characteristics are summarized in Table 2.

**Table 2:** Patient characteristics of our institutional data

Patient characteristics	
Median age at diagnosis	53 (20–65)
*Sex*	
Female	31 (58.5%)
Male	22 (41.5%)
*Location*	
Parietooccipital	3 (5.7%)
Temporal	18 (34%)
Frontal	15 (28.3%)
Temporooccipital	3 (5.7%)
Temporoparietal	4 (7.5%)
Parietal	7 (13.2%)
Occipital	3 (5.7%)
*Tumor side*	
Right	32 (60.4%)
Left	21 (39.6%)
*Extent of resection for re-resection*	
GTR	32 (60.4%)
STR	21 (39.6%)
*Extent of resection for first resection*	
GTR	34 (64.2%)
STR	19 (35.8%)
Median time between first and re-resection in months	7 (6–16.5.5)
*MGMT promotor status*	
Methylated	26 (49.1%)
Not methylated	27 (50.9%)
*Postoperative complications after the re-resection*	
Wound healing deficit	3/53 (5.7%)
Cerebrospinal fluid fistula	2/53 (3.8%)
*Postoperative complication after the first resection*	
Wound healing deficit	2/53 (3.8%)
Cerebrospinal fluid fistula	1/53 (1.9%)
*Contrast enhancement at recurrence*	
Yes	53/53 (100.0%)
No	0/53 (0.0%)
*Intraoperative MRI*	
Yes	12/53 (22.6%)
No	41/53 (77.4%)
*Adjuvant therapy*	
Radiochemotherapy	30/53 (56.6%)
Radiotherapy	4/53 (7.5%)
Chemotherapy	2/53 (3.8%)
No therapy	17/53 (32.1%)

### Progression-free survival and overall survival in wild-type glioblastoma of our institutional data

A total of 53 patients were included from our institutional cohort. The median age of the patients was 53 (Interquartile Range (IQR): 20–65). The median time between first resection and re-resection was 7 months (IQR: 6–16.5). Among the 53 patients, 31(58.5%) were female and 22 (41.5%) were male. Tumor locations were located in the temporal (34%), frontal (28.3%), parietal (13.2%), temporoparietal (7.5%), parietooccipital (5.7%), temporooccipital (5.7%), and occipital (5.7%) regions. Tumors were more frequently located in the right hemisphere (60.4%) compared to the left (39.6%). GTR was achieved in 34 (64.2%) patients and STR in 19 (35.8%) patients at the time of the first resection, compared to 32 patients (60.4%) with GTR and 21 (39.6%) patients with STR at time of the re-resection. MGMT promoter hypermethylation was present in 26 (49.1%) of patients. Wound healing deficits occurred in 2 (3.8%) patients and cerebrospinal fluid (CSF) fistula in 1 (1.9%) patient after the first resection. Following re-resection, 3 (5.7%) of patients experienced a wound healing deficits and 2 (3.8%) developed CSF fistula. Due to these complications, additional neurosurgical interventions were required in these cases. Intraoperative MRI in re-resection for recurrent GB was performed in 12 (22.6%) patients. As a part of adjuvant therapy for recurrent GB, 30 (56.6%) patients received radiochemotherapy, 4 (7.5%) patients received radiotherapy alone, 2 (3.8%) patients received chemotherapy alone. Among 53 patients, 17 did not receive any further adjuvant therapy.

Among 53 patients with available PFS data, 32 (60.4%) patients underwent re-resection with CE-RTV ≤ 1 cm³, while 21 (39.6%) underwent re-resection with CE-RTV > 1 cm³. The mean PFS in months for CE-RTV ≤ 1 cm³ cohort was 6.6 months (95% CI: 2.3–11.0), compared to 5.8 months (95% CI:0.3–12.0) for the CE-RTV > 1 cm³ cohort. The difference in PFS between the two groups was not statistically significant *(p* = 0.529). For OS, data from all 53 were analyzed. The mean OS following CE-RTV ≤ 1 cm^3^ was 11.4 months (95% CI: 6.3–16.5) whereas the mean OS for the CE-RTV > 1 cm³ cohort was 5.5 months (95% CI: 3.2–7.7). The difference in OS between the EoR groups was not statistically significant (*p* = 0.229). A summary of the patient characteristics of our institutional data is presented in Table 2.

### Progression-free survival and overall survival in recurrent wild-type glioblastoma after re-resection: pooled analysis with both institutional and IPD

**Fig. 2:**
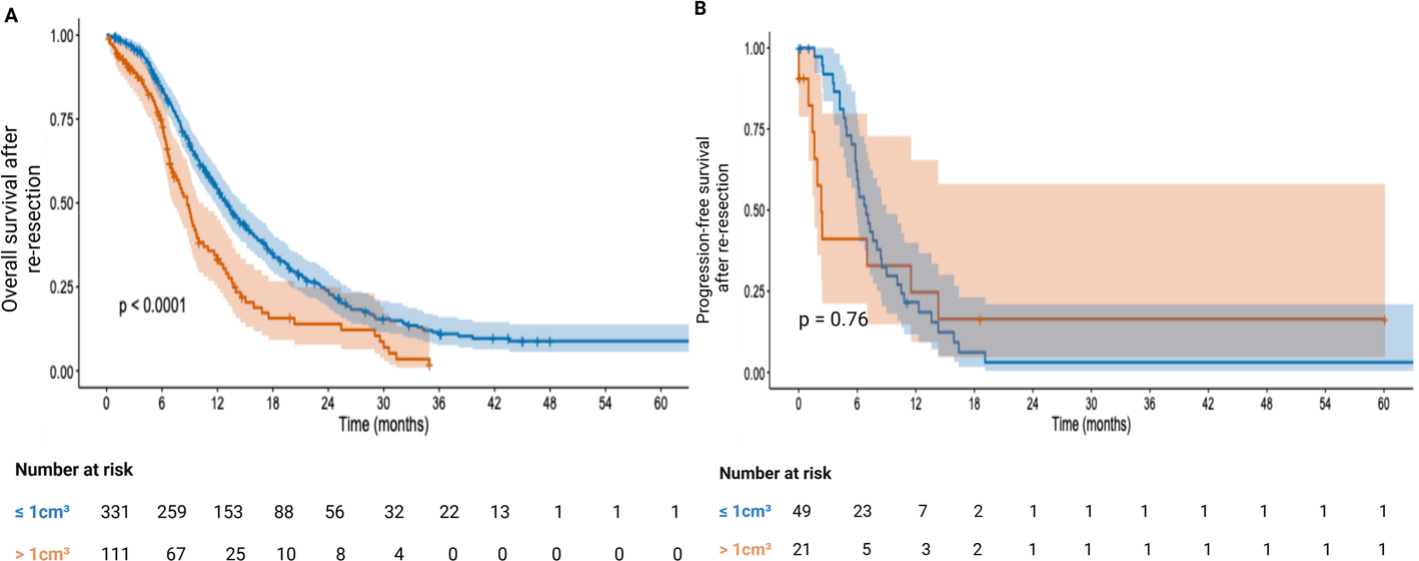
Kaplan-Meier survival analysis for progression-free survival (PFS) and overall survival (OS) based on the extent of resection (EoR) after re-resection, where EoR was stratifiied as a contrast-enhancing (CE) residual tumor volume (RTV) of ≤ 1 cm³ or > 1 cm³. The analysis included 442 patients for OS after re-resection based and 70 patients for PFS after re-resection. **A, B** CE-RTV ≤ 1 cm^3^ is represented by an orange line and CE-RTV > 1 cm^3^ by a blue line. Patients at number-at-risk at defined times in months were added below the Kaplan-Meier survival curves. Shaded areas in the Kaplan-Meier survival curves represent the 95% Confidence Interval (CI) areas. A: Mean OS after re-resection was 14.4 months (95% CI: 13.2–15.6) for CE-RTV ≤ 1 cm³ and 8.8 months (95% CI: 7.4–10.2) for CE-RTV > 1 cm³. The Kaplan-Meier curve for OS after re-resection showed a statistically significant difference based on EoR (*p* < 0.0001) whereas B: Mean PFS after re-resection was 7.2 months (95% CI: 4.3–10.0) for CE-RTV ≤ 1 cm³ and 5.8 months (95% CI: 0.3–12.0) for CE-RTV > 1 cm³. There was no statistically significant difference in PFS based on EoR after re-resection in 70 patients (*p* = 0.76)

A total of 442 IPD were available for OS analysis after re-resection, with 53 were from our own institutional data. Among them, 331 (72.2%) patients underwent re-resection with CE-RTV ≤ 1 cm^3^, while 111 (27.8%) underwent re-resection with CE-RTV > 1 cm^3^. The median OS after re-resection in months for CE-RTV ≤ 1 cm^3^ cohort was 14.4 months (95% CI: 13.2–15.6), compared to 8.8 months (95% CI: 7.4–10.2) for the CE-RTV > 1 cm^3^ cohort. The difference in OS after re-resection between these groups based on EoR was statistically significant (HR: 1.731, 95% CI: 1.342–2.234), *p* < 0.0001). For PFS, institutional data and PFS data after re-resection from Bao et al. (17) were used to generate a Kaplan-Meier survival curve. A total of 70 patients were included in the analysis with 49 (70%) patients undergoing re-resection with CE-RTV ≤ 1 cm^3^ and 21 (30%) patients with CE-RTV > 1 cm^3^. The mean PFS after re-resection in CE-RTV ≤ 1 cm^3^ group was 7.2 months (95% CI: 4.3–10.0) while in CE-RTV > 1 cm^3^ group, it was 5.8 months (0.3–12.0). There was no statistically significant difference in PFS after re-resection between the groups based on EoR (*p* = 0.76). The Kaplan-Meier survival curves for OS and PFS after re-resection are shown in Fig. 2.

### Bias and quality evaluation

The NIH Quality Assessment for retrospective studies evaluated four studies: Karschnia et al. [15], Kim et al. [13], Straube et al. [12], and Bao et al. [14]. All studies clearly stated their research objectives, defined their study populations, and ensured that at least 50% of eligible participants were included. They also provided information on subject recruitment and eligibility criteria, measured exposures before outcomes, and used a sufficient time frame for analysis and therapy monitoring. Furthermore, they examined different levels of exposure in relation to outcomes, defined and validated exposure measures, and clearly specified outcome measures. Additionally, all studies accounted for loss to follow-up (≤ 20%) and adjusted for key confounding variables using statistical measures.

Taken together, the NIH-QAT indicated moderate methodological quality across studies with notable limitations. In particular, none of the studies justified sample size, exposures were assessed only once, and blinding was not used, which increases the risk of bias. Table 3 presents the NIH Quality assessment of the studies which were included in the meta-analysis for their methodological accuracy based on specific NIH criteria. Each study is assessed on various aspects for including the clarity of research question, the definition of the study population, adequacy of patient follow-up and consideration of other confounding factors. Studies are classified as “Yes” if they meet a criterion, “No” if they do not.

**Table 3:** NIH quality assessment for retrospective studies

Criterions	Karschnia et al. [15], 2023	Kim et al. [13], 2024	Straube et al. [12], 2024	Bao et al. [14], 2024
Research question or objective stated	Yes	Yes	Yes	Yes
Study population defined	Yes	Yes	Yes	Yes
Participant rate at least 50% from eligible	Yes	Yes	Yes	Yes
Subject recruitment and eligibility criteria	Yes	Yes	Yes	Yes
Sample size justification	No	No	No	No
Exposure(s) measured prior to the outcome(s)	Yes	Yes	Yes	Yes
Sufficient time frame	Yes	Yes	Yes	Yes
Examination of different levels of exposure as related to the outcome	Yes	Yes	Yes	Yes
Definition and validation of the exposure measures	Yes	Yes	Yes	Yes
Exposure(s) assessed more than once	No	No	No	No
Definition of outcome measures	Yes	Yes	Yes	Yes
Blinded assessors	No	No	No	No
Loss to follow-up after baseline of 20% or less	Yes	Yes	Yes	Yes
Statistical measure and adjustment of key confounding variables	Yes	Yes	Yes	Yes

## Discussion

The aim of the present study was to investigate the impact of EoR in recurrent GB on PFS and OS after re-resection. The EoR was defined as CE-RTV ≤ 1 cm^3^ and CE-RTV >1 cm^3^ after re-resection. To answer this question, we performed a pooled meta-analysis. Additionally, institutional data was also added to the present work. In our institutional data with 53 patients following re-resection, no statistically significant difference in PFS was observed based on EoR (*p* = 0.529). Similarly, OS after re-resection did not significantly differ according to EoR (*p* = 0.229). The pooled results with 442 patients showed that patients who underwent re-resection with CE-RTV ≤ 1cm^3^ compared to the CE-RTV >1 cm^3^ group, had improved OS after re-resection, with a median of 14.4 months vs. 8.8 months, respectively (*p* >0.0001). Additionally, our findings showed that patients who underwent re-resection with a postoperative CE-RTV of >1 cm³ had a higher risk of reduced overall survival (OS) after re-resection compared to those with a postoperative CE-RTV of ≤ 1 cm³ (HR: 1.731, 95% CI: 1.342–2.234, *p* < 0.0001). The analysis of EoR regarding PFS of 70 patients after re-resection, compared to STR in our institutional data and the study by Bao et al., showed no statistically significant differences between these groups (HR: 1.115, 95% CI: 0.559–2.223, *p* < 0.76). Consistent with prior evidence, a large single-center cohort showed that both the initial and repeat extent of resection stratify overall survival, and that achieving gross total resection at recurrence yields the most favorable overall survival, mitigating the adverse effect of an initial subtotal resection and suggesting that patients initially treated with subtotal resection may still benefit from re-resection to gross total resection when safely feasible [20]. 

Within the pooled dataset, the study results reported by Karschnia et al. are consistent with the association we observe, that is, longer survival for RANO class 1–2 versus class 3 resections. RANO 1 is defined as supramaximal resection with complete resection of CE tumor volume, while RANO 2 is defined as maximal resection, characterized by a CE-RTV resection of ≤ 1 cm³. In contrast, RANO 3 is defined as submaximal resection with CE-RTV >1 cm³ [15]. Within the RANO resect framework at re-resection, our CE-RTV based dichotomy (≤ 1 cm³ vs. >1 cm³) maps to the contrast enhancing component of the RANO strata, with ≤ 1 cm³ approximating Class 1–2 and >1 cm³ corresponding to Class 3. Non-contrast-enhancing abnormality was not used to define EoR in our institutional series and is therefore outside the scope of our classification. Kim et al. also showed an improved postprogression survival (PPS) of GTR compared to non-GTR group [13]. Another study from Honeyman et al. showed in 83 patients, where EoR was defined as >95% and < 95% also an improved OS without significant difference in overall complication rates between primary versus secondary or third-time resections [21]. 

While the treatment strategies in primary IDH-wildtype GB are clearly defined with neurosurgical resection followed by radiochemotherapy, there is a lack for standardized treatment recommendations for recurrent GB, which leads to a difficult decision-making process [22, 23]. In clinical practice therapy options in recurrent GB vary between re-resection, re-radio-chemotherapy, experimental approaches such immunotherapy and tumor-treating fields (TTF) [24–26]. The lack of guidelines and limited evidence from randomized studies hamper the decision on the accurate therapy strategy. Our findings support the findings from Karschnia et al. [15] that re-resection with a postoperative CE RTV ≤ 1 cm^3^ in recurrent IDH-wildtype GB leads to statistically significant improvement in survival after re-resection compared with a resection with >1 cm^3^ CE-RTV. However, it is not clear which patients benefit most and how it is to be combined with other therapeutic approaches.

While our findings reinforce the role of maximal safe resection in recurrent GB, additional factors must be considered to refine neurosurgical decision-making. Future research on recurrent GB with primary data should concentrate on molecular markers in recurrent GB patients. MGMT promoter methylation, CDKN2A/B, TERT, EGFR and PTEN, as well as other genetic mutations might influence PFS and survival after re-resection [27–29]. Additionally, the promising approach of supratotal resection techniques (e.g., FLAIRectomy, lobectomy, resection margins) in surgery for primary tumors will have to be further validated for recurrent GB [30–32]. Recent studies have underscored the value of supramaximal resection, including the resection of non-CE FLAIR abnormalities commonly referred to as FLAIRectomy. While this concept has primarily been applied to newly diagnosed GB, removal of infiltrative tissue beyond the enhancing margins has been associated with improved survival outcomes in select patients, although at the potential cost of increased morbidity [15]. The RANO classification incorporates FLAIR components into Class 1 resections, defined as 0 cm^3^ CE tumor and ≤ 5 cm^3^ non-CE tumor. However, supramaximal RANO class 1 resections are not superior to RANO class in the context of second surgery for recurrent GB. ^15^ However, re-resection of recurrent GB can be challenging because intraoperative differentiation of tumor and healthy tissue is more challenging and 5-ALA guided surgery has a lower specificity and negative predictive value in recurrent surgery [33, 34]. 

Furthermore, there is a need for randomized studies on combination therapy with TTF, immunotherapy and targeted radiotherapy after re-resection. Finally, it is essential to integrate postoperative QoL after re-resection into the studies as a key endpoint to ensure that prolonged OS is not associated with severe neurological deficits. 

## Limitations

The present study also has several limitations. The retrospective nature of the present study and the included studies can lead to a selection bias. Although we applied consecutive inclusion, surgical decision-making inherently favors fitter patients (e.g. higher KPS, operable location), which can introduce selection bias that cannot be fully eliminated in a retrospective design. A key limitation is that, beyond EoR, MGMT status, tumor location, time to re-resection, and post-re-resection therapy could not be incorporated into the survival models because harmonized individual-level data were unavailable across the contributing studies, and no IPD-capable KM curves stratified by EoR in combination with these variables were reported. As a result, our pooled analyses were restricted to EoR and potential residual confounding by these factors cannot be excluded. The findings should be interpreted within this analytic scope. Another limiting factor is the lack of analysis of tumor localization and its influence on the EoR with the associated influence on OS and PFS. Furthermore, there might be a small heterogeneity regarding the adjuvant treatment regimen after re-resection. Across the included external series, virtually all patients underwent adjuvant radiochemotherapy after re-resection (Kim et al., Straube et al., Bao et al.: 100%; Karschnia et al.: 91.5%), whereas our institutional cohort was more heterogeneous (56.6% radiochemotherapy, 7.5% radiotherapy alone, 3.8% chemotherapy alone, 32.1% none). We now explicitly discuss this imbalance as a potential confounder and a limitation of cross-series comparability. Future large-scale investigations should assess whether the association between extent of resection and outcome varies across molecular backgrounds. In primary glioblastoma, Roder et al. [34] demonstrated in a prospective multicenter trial that achieving 0 cm³ contrast-enhancing residual tumor is associated with improved PFS and OS, and that the adverse effect of any residual (> 0 cm³) is most pronounced in MGMT-unmethylated tumors, with no significant OS difference between 0 cm³ and > 0 cm³ in MGMT-methylated tumors. Since resectability depends heavily on proximity to eloquent brain regions, this could be a relevant confounder. An important limitation is heterogeneity in EoR definitions. Bao et al. [14] reported only patients with GTR at second surgery and did not publish postoperative CE-RTV volumes. Kim et al. [13] defined GTR as > 99% removal on early postoperative MRI rather than by an absolute residual volume. In the latter, a residual > 1 cm³ would arithmetically require a preoperative CE volume ≥ 100 cm³. Given that preoperative re-resection volumes in contemporary series (e.g., Bao et al. [14]) typically range around 26–62 cm³, such residuals are unlikely in most > 99% GTR cases, although we cannot exclude rare edge cases due to unavailable per-patient CE-RTV. Consequently, mapping GTR to CE-RTV ≤ 1 cm³ may introduce non-differential misclassification in these cohorts. Assertions about a CE-RTV ≤ 1 cm³ target are therefore most directly supported by volumetric/RANO-classified cohorts (Karschnia et al. [15] and institutional data) and should be viewed as pragmatic guidance rather than a universally validated cut-off across all included datasets. Although the likelihood of recurrent GB exceeding a size of 100 cm^3^ is low, it cannot be completely excluded. In cases where GTR was reported, we defined that the CE-RTV was ≤ 1 cm^3^. Finally, another important aspect and limitation is that the quality of life (QoL) after re-resections was not able to be taken into this analysis. Although an improved survival was found in postoperative CE-RTV of ≤ 1 cm³ re-resection compared to CE-RTV of > 1 cm³, data on the postoperative neurological outcome and the associated influence on the QoL of patients are missing.

## Conclusions

In conclusion, in the present study we investigated the impact of re-resection and the role of EoR in recurrent IDH-wildtype GB surgery regarding overall survival after re-resection and PFS. The results of this study confirm that a RTV ≤ 1 cm^3^ enhances the survival time after diagnosis of recurrent GB compared to a CE-RTV > 1 cm^3^ in recurrent wild-type GB. Standardized guidelines to optimize treatment strategies for recurrent IDH-wildtype GB are needed.

## Electronic Supplementary Material

Below is the link to the electronic supplementary material.

Additional file 1.

## Data Availability

The institutional clinical data generated and analyzed during this study are available from the corresponding author upon reasonable request. The individual patient data (IPD) used in the meta-analysis were reconstructed from publicly available Kaplan–Meier curves of published studies, following established methodology. The systematic review protocol was registered in PROSPERO under CRD420250654313. https://www.crd.york.ac.uk/prospero/display_record.php?ID=CRD420250654313.
