# Supplementary figures and images for: Extent of reoperation predicts survival in recurrent IDH-wildtype glioblastoma based on institutional data and individual patient data meta analysis
Source: Discov Oncol. 2025 Nov 13;16:2099. doi: 10.1007/s12672-025-03928-8 (PMC12615886; doi:10.1007/s12672-025-03928-8)

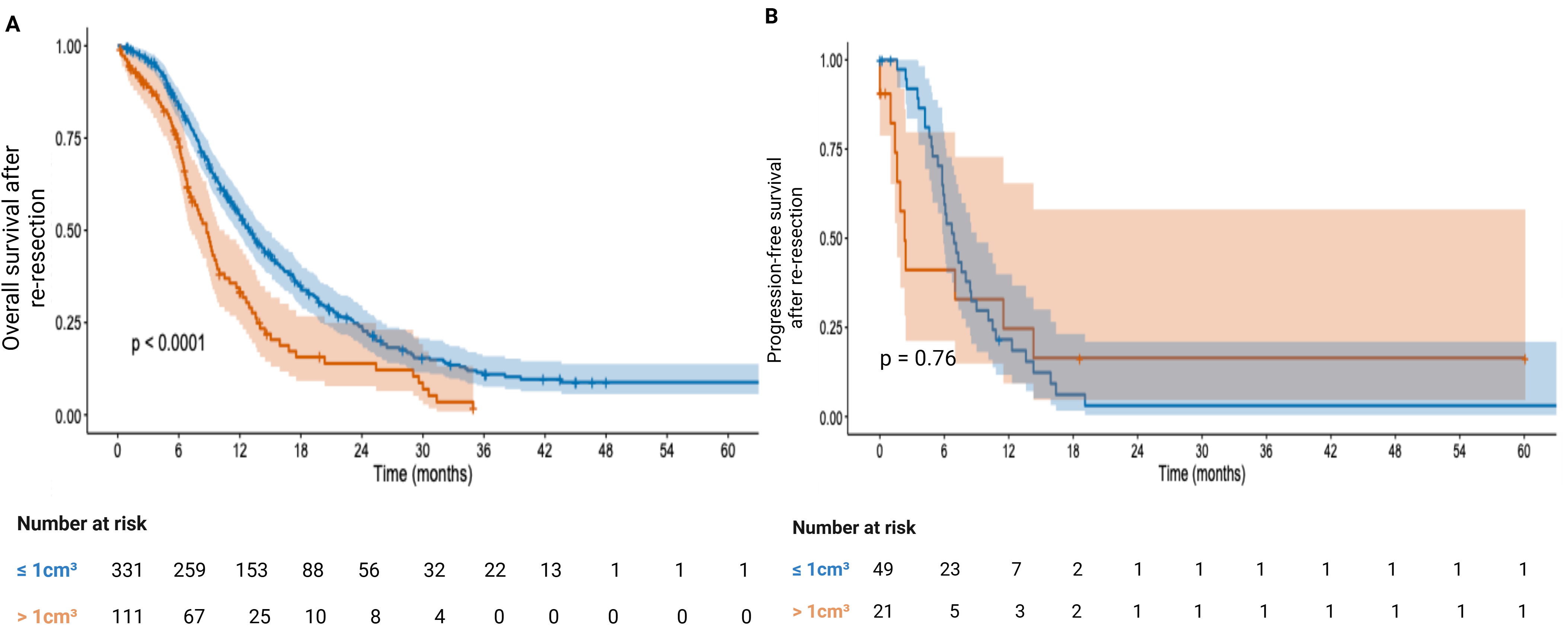

Supplement: Supplementary file 1 — Additional file 1. [file 12672_2025_3928_MOESM1_ESM.png]
